# Diet, Physical Activity, Weight Status, and Culture in a Sample of Children from the Developing World

**DOI:** 10.1155/2012/242875

**Published:** 2012-10-30

**Authors:** Pamela S. Gaskin, Pamela Lai, Devon Guy, JaDon Knight, Maria Jackson, Anders L. Nielsen

**Affiliations:** ^1^Faculty of Medical Sciences, University of the West Indies, Martindale's Road, St. Michael, Cave Hill, Barbados; ^2^School of Dietetics and Human Nutrition, McGill University, Montred, QC, Canada H3A 0G4; ^3^Faculty of Medical Sciences, University of the West Indies, Mona, Jamaica

## Abstract

*Objective*. Barbados, a small developing state at the end of the nutrition transition, faces an obesity epidemic. Although there is hope of stemming the epidemic in childhood, no descriptions of children's dietary and physical activity (PA) patterns are available for planning purposes. We describe the food and activity preferences and adult encouragement of active and sedentary behaviors for children 9–11 years in relation to weight status and the cultural context. *Design*. We used data from a pilot study preceding a large-scale ongoing study on the local drivers of the obesity epidemic among preadolescent children. PA, sedentary activity, and dietary intakes were assessed from recalls. Weight and height were measured. *Setting*. Barbados. *Subjects*. Sixty-two (62), 9–11-year-old school children. *Results*. Sugar-sweetened beverages provided 21% of energy consumed. Energy intake significantly explained BMI. Parents selected significantly more of children's sedentary activities and encouraged mostly homework and chores (59%). Children's self-selected school-based activity was significantly related to BMI. *Conclusions*. Childhood obesity prevention recommendations and research should focus on culture-specific practices that promote acquired taste for excess sugar and parent-child interactions regarding PA. Child influenced by school-based activity intervention may an important area for preventive intervention research.

## 1. Introduction

Barbados is a small developing nation with a tourist driven economy. It is at the end of the nutrition transition. Despite recent economic challenges, it has a developed world profile of chronic metabolic disease with especially high levels of diabetes [1] and hypertension [2]. Amongst this predominantly African origin population, more than 60% of adults over 40 years are overweight or obese since the early 1990's [2]. Gaskin et al. estimated overweight prevalence at 27% among adolescents more than a decade ago [3]. These adolescents had inadequate knowledge about appropriate food, body size, and levels of activity. The last nationally representative data collected on child weight status in Barbados dates from 1981 and estimated that less than 5% of children were overweight [4]. The most recent preliminary estimate for children 9-10 years puts prevalence of overweight at 35% [5]; this adds to the evidence that there is an emergent childhood obesity epidemic in Barbados. Importantly there are no comprehensive dietary intake or activity studies on this critical preadolescent age group who are at a stage in the life cycle where intervention is likely to have a large impact on the entire cascade of metabolic disease. Furthermore there is evidence that energy and fat consumption have increased steadily in the recent past [6] as well as intake of sugar among adults [6]. Physical activity among children is likely to be low as suggested by a school-based study which reported low participation in physical education among primary and secondary school students [7]. 

Diet, physical activity, and sedentary behaviours are key contributors to energy balance and lend themselves to meaningful obesity interventions especially in childhood. However these interventions must be informed on the factors that are unique to the locale and those that mediate major obesity risk factors in children. 

School therefore plays an important role in the childhood environment as it relates to energy balance in Barbados. Eighty one (81%) percent of eligible children attend public primary schools, and The Barbados School Meals Service provides lunches (school lunch) prepared at centres and delivered to the schools on a daily basis. On a given day a school receives one meal from a set menu, no drinks are included, and no options are offered. Formal physical education (PE) classes are offered in the school curriculum. Nevertheless it is well established that parents transmit strong culture-based beliefs that influence children's food intake and activity [8]. In the Barbados setting, there is cultural memory of food scarcity and debilitating labor associated with a post slavery experience [9], that anecdotally appears to promote excessive offerings of food to children and may underlie negative attitudes to physical activity. These factors make understanding the nuance in dietary and active behaviours critical. In addition, there are no specific national child centred obesity programs, and the recently published local nutrition recommendations do not include recommendations for the preadolescent age group. At present, education for the general public on physical activity (PA) and sedentary behaviours such as the reduction of screen time (time spent on television/video games/computer) is promoted in an ad hoc manner. Since diet, activity, and sedentary behaviours are all associated with weight gain and may all be affected by culture [10], and examination of these factors among children in the local context is necessary to inform the relevance and application of obesity prevention recommendations. The exploration of the role that parents, or other adults in the home, play in children's active behaviors is warranted.

In this study, we examined the dietary intakes, active physical and sedentary, activity of children 9–11 years. Our aim was to describe the food and activity preferences and adult encouragement of children's active and sedentary behaviors and further to relate these patterns to the cultural context. 

## 2. Materials and Methods

The sample was a convenience sample of 29 boys and 33 girls 9–11 years old who attended a public primary school in Barbados. They were chosen to test the methods for a study intended to estimate the prevalence of overweight and obesity in this age group. The study was approved by the Institutional Review Board of the University of the West Indies/Ministry of Health, Barbados. Parents and children gave written and informed consent to participate in this study.

### 2.1. Measurements

#### 2.1.1. Anthropometry

Two consecutive measurements were performed for each anthropometric index. A third measure was taken if a difference of more than 0.5 cm or 0.5 kg was observed. Standing height was measured to the nearest 0.1 cm following standard procedures [11]. Height was measured without shoes on a floor-standing stadiometer (Charder Co. Ltd., HM200P, USA), to the nearest 0.1 cm. Reliabilities for standing height were performed prior to data collection. Body weight was measured by digital scale (Taylor 7331 Digital Scale, USA) using a standard protocol to the nearest 0.1 kg [11]. The scale was calibrated daily. Body mass index (BMI) was calculated (weight (kg)/height^2^ (m^2^)).

#### 2.1.2. Dietary Repeated Measurements of 24-Hour Recalls

Three 24-hour recalls were administered on nonconsecutive days that included 2 weekdays and 1 weekend day. Two trained researchers collected data on 24-hour recalls, and students were unaware of the days the interview would be conducted. The interviewers requested participants to recall all food and drink consumed over the previous 24 hours. Portions were carefully estimated by use of food models (Nasco, USA), household measures, and utensils in conjunction with a detailed description of the food and method of preparation. We compared intake to the acceptable macronutrient distribution ranges (AMDR) [12], to RDA for the Caribbean countries [13] and to the food-based dietary guidelines for Barbados [14].

#### 2.1.3. Physical Activity Measurements Repeated for 24-Hour Recalls

Physical activity recalls were conducted on nonconsecutive days and included 2 weekdays and 1 weekend day. Physical activity data were collected using both 2, 24-hour recalls and 1 weekly activity recall. Twenty-four-hour activity recalls consisted of a table allowing observers to fill in prior day's activities in a manner similar to that used on the 24-hour dietary recalls, their location (home/school), activity level (sedentary or active), and whether the activity was self-selected or adult-guided. Information collected included whether the child has space to play outside their home. Weekly activity recall forms were similar to the 24-hour activity recalls, but included a column to record the number of days per week specific activities occurred. All activities occurring at least once weekly were recorded and listed activities were classified as active or sedentary based on previously developed guidelines [15]. For example, physical education class was coded as active and homework sedentary. Mode of transportation to school (active or sedentary) was included in each student's list. Frequency of active and sedentary counts were obtained and used to determine activity pattern. Data from weekly activity recalls was not used in calculations. 

### 2.2. Statistical Analysis

Children's weight status was categorized using the International Obesity Task Force (IOTF) BMI cutoff points [16]. Nutrition analyses were completed using Nutritionist Pro Diet Analysis (Version 4.3.0, Axxya Systems, USA). 

Differences in proportions were examined using Chi-squared or Fisher's exact test and Student's *t*-test used for continuous data. Relationships between dietary and activity variables and BMI were first examined using Pearson Product Moment correlation analyses. The influence of dietary intake and physical activity on BMI was examined by linear regression analyses. In all multivariable models, adjustments were made for potential confounders and included age and sex. Logistic regression analyses were performed to test whether diet or activity was associated with weight status. Data were analyzed using SPSS (version 19, IBM, USA). Statistical significance was achieved when *P* < 0.05.

## 3. Results

### 3.1. Characteristics of Participants

 Among the 62 predominantly African children (29 boys, 33 girls), mean age was (boys 10.25 ± 0.60 years; girls 10.06 ± 0.65 years). Boys tended to be heavier and have higher BMI but this was not statically significant. Mean weight was (boys 43.9 ± 13.92 kg; girls 39.73 ± 11.81 kg), height (boys 144.89 ± 6.43 cm; girls 145.11 ± 7.09 cm), and BMI (boys 20.70 ± 5.84 kgm^−2^; girls 18.71 ± 4.61 kgm^−2^). The prevalence of overweight was 37.1% (95% CI 24.7; 49.4) and did not differ significantly by sex. Thirteen (45%) boys and 10 (30%) girls were overweight. One-half of the children walked to school while 42% took the bus and 8% arrived by car. More than 80% of children ate school lunch in addition to a snack or drink bought at or brought to school. More than 75% of children reported that they had a place outside to play at home.

### 3.2. Nutrient Analysis

The average daily energy intake of the children was 7806 ± 1793 kJ (1865 ± 428 kcal); for boys 8441 ± 1829 kJ (2017 ± 436 kcal); for girls 7248 ± 1587 kJ (1732 ± 397 kcal). Boys consumed significantly more energy, proteins, total fat, saturated fat, sodium, calcium, and iron than girls (*P* < 0.05). The distributions of carbohydrates, protein, and fat are within the acceptable macronutrient distribution ranges (AMDR) (Table 1). Vitamin D intake was low in both sexes.

Except for energy intake, the nutrient profile of the subjects varied considerably from the Caribbean recommendations. The mean daily percentage of energy from protein and fat were high and intake from saturated fat was at the upper end of the recommended range [13] (Table 2).

### 3.3. The School and Food Availability

Most children ate a snack at mid-morning which consisted of either sugar-sweetened milk provided by The School Meals Department of the Ministry of Education and Human Resource Development Barbados, (The Barbados School Meals Service), or a sugar-sweetened beverage purchased at the school or packed from home. At midday, approximately 80% of children ate the government provided school lunch usually supplemented with a sugar-sweetened beverage purchased on the school compound or packed from home. Some snack items were available in the environs of the school but were usually purchased prior to or after school hours. 

Table 3 shows the nutrient composition of a sample school lunch compared with the nutrient composition of a sample lunch taken from the food-based dietary guidelines for Barbados [17]. The sodium content of the sample school lunch was very high.

### 3.4. Top Foods and Beverages Consumed

Sugar-added beverages accounted for 21% of the energy consumed and dominated the list of the top 20 most frequently consumed foods (2.6 servings per day); this included colloquial drinks and homemade hot chocolate (0.65 servings per day). Drinks were mostly fruit juice or juice like drinks (1.5 servings per day) and rarely cola flavored aerated beverages (0.03 servings per day) (Table 4). Frequently consumed colloquial drinks and flavoured milks had high energy content per serving (per 250 mL serving mauby −558 kJ ( kcal); Tutti Frutti (flavoured milk) −839 kJ (200 kcal)).

### 3.5. Relationships of Body Composition to Dietary Intake

As BMI increased so did energy intake, the macronutrients as well as calcium and iron. In linear regression controlled for age and sex, with the exception of carbohydrate, BMI continued to be positively related to the macronutrients although the relationship with carbohydrates lost statistical significance. Mean energy intake adjusted for age and BMI was 999 kJ ± (SE 435) (238 kcal ± (SE104)) higher among boys than among girls. 

### 3.6. Activity

The range of active activities were those commonly reported for this age group. These included football, cricket, “run-about,” walking, weekly physical education class, hand games, dancing, ball games, tag, hide and seek, hop scotch, and household chores. Frequently reported sedentary activities included watching television, playing video or computer games, doing homework, playing board games and card games, and receiving tutoring or music lessons. Screen time accounted for 21% of children's activities. Homework and chores were predominately adult-selected and accounted for 59% (33% and 26%, resp.) of adult selected activities, and 26% of all activities. Children reported watching less television on weekdays than on weekends. Walking as a means of travel accounted for 5% of reported activities. Overall children selected more of both sedentary and active activities than parents but the difference was larger for active activities (Table 5).

### 3.7. Relationships of BMI to Dietary Intakes and Activity

Children's self-selected activities at school were significantly and negatively correlated with BMI (*P* < 0.05); no other measures of activity neither sedentary nor active, as assessed by either weekly or 24-hour recalls were significantly related to BMI or weight status adjusted for age and sex. In regression analysis, energy intake and self-selected active activities at school, adjusted for age and sex, explained 18.4% of variance in BMI (Table 6). 

In logistic regression, children of normal weight consumed less energy and were 3.9 (95% CI 10.89, 17.52) more likely to engage in self-selected school-based active activities than overweight children.

## 4. Discussion 

The general characteristics of the sample in terms of race profile, availability of money to purchase snacks, and some travel to school by bus and car suggest a socioeconomic status mix similar to general population which has a large middle class. The adult population is 93% black and per capita gross national product $23,700.00, with a negligible percentage living in poverty [18]. This translates to wide access to calories.Thirty seven percent (37%) of the children 9–11 years in our pilot study were overweight and this was similar to the 35.6% (95% CI 31.6, 39.6) preliminary estimate [5]. The mean daily percentage of energy from protein and fat was high and intake from saturated fat was at the upper end of the recommended range. These patterns were nevertheless quite similar to those reported by Sharma et al. from study using food diaries among adults in this population [6]. 

The children's diets are in keeping with patterns seen worldwide that accompany increasing obesity. This population may be at greater risk given that blacks in other settings have higher levels of overweight [19] and had larger secular increases in childhood obesity than other races [20]. 

Sugar-sweetened beverages (1.5 servings per day) provided 21% of the calories consumed. This may represent misinformation on nutrient contents of foods [21] and adherence to cultural norms [6] and is likely to predispose susceptible children to overweight. The patterns are quite similar to reported intakes among adults in this population [6]. This points to the influence of parents dietary habits on children's food intake at this age, reinforcing the importance for preadolescent children of adult modeling [22]. 

The pattern of drink consumption coincides with notions of juices and local drinks as “healthy;” lack of information and recommendations on locally produced foods exacerbates this situation. It should be noted that the current Barbados dietary guidelines do not have recommendations for children below age 14 years so that the 1993 Caribbean Food and Nutrition Institute recommendations [13] are the sole guide for this age group. These are dated and assume that Caribbean children will not be subject to pressure of weight gain so the calorie allowances are higher than for children in developed countries [13]. Flavoured milk, a sugar-added beverage was frequently reported and is different from milk consumed by children in countries like the USA and Canada where unsweetened milk is consumed. The acquired taste for sugar is an area of particular importance in our cultural context where sugar production remains important in the economy and likely originates from olden times when, for example, colloquial sugar-added beverages drinks, like Mauby, were sold to cane field workers, at low cost. In the modern context, these drinks are likely to promote weight gain. 

Most of the children in our study ate lunch from the school meals service. When we compared a sample lunch to guidelines, the energy content fell within one-third of daily requirements and the dietary reference intakes for Canada [12] and the estimated calorie needs per day for the USDA [23]. It would theoretically provide 34% calories from fat which exceeds the 30% upper limit recommended. It should be borne in mind that children often purchased a sugar-sweetened beverage to accompany lunch, which would put the meal outside of the desirable range of calories. By comparison the sample meal from the Barbados guidelines [17] was more balanced and would provide 22% calories from fat. The high level of sodium intake reported by the children corresponded to the high sodium content of the sample school lunch. These findings highlight the need for assessment of the nutrient content of meals served by Barbados School Meals Service in view of the high uptake of lunches. 

Our results showed that children's self-selected school-based activities were the only active measures which correlated with BMI. This may be protecting against weight gain or may represent children who are slimmer and more confident about physical activity. Schools may therefore provide a premium opportunity to promote enjoyable activity that is sustained via personal choice. It is clearly important to understand how self-selection of activities impinges upon propensity to engage in a sustained manner among these preadolescent children. We were unable to find any study that examined associations with the self-selected as opposed to adult guided activities. These considerations may be useful in the promotion of active behaviors in children. 

Few children reported parental encouragement of active activities or activities that might be considered pleasurable. This may have origins in tradition, for instance, many women are unable to swim despite the proximity of excellent beaches, so they in turn cannot model swimming. It is likely that many parents do not think they need to play a role in promoting pleasurable activity. Parents guided activities tended to be mostly homework and chores (59%); historically formal education was seen as the single route to success for the African population in Barbados. The emphasis on homework and high reporting of extra lessons reflect the premium placed on academic achievement for Barbadian children. Minimal parental involvement in promotion of enjoyable activities is of concern as parental modeling, and encouragement behaviors are known to be very important in this age group [22].

The high reporting of screen time is important as this sedentary type of activity is associated with weight gain [24]. We as we did not see a significant relationship of BMI to sedentary behaviours, their frequency of reporting is nevertheless a cause for concern. It is likely that the cross-sectional nature of the study would mask relationships to weight change [25]. We were surprised at the low level of activities reported at school. This was likely exacerbated by the intended omission of regular class room reading and writing from recalls which would reduce the reported sedentary activities at school. PE would be the most reported active activity but formal classes were held only once per week. This school did not have its own playground which may have caused them to have fewer activities than usual. Also after school, active activities would tend to involve the few athletes who play team sports. Nevertheless, the low number of active activities at school is in line with Proshaks's findings among older Barbadian when most children reported the majority of their physical activity took place after school (58%) [7].

### 4.1. Limitations

We are aware of the limitation of the using 24-hour recalls; however, these are the most commonly used method of dietary data collection through self-reports [26] and has been shown to be more accurate when it is closer in time to the interview. As such, 24-hour recalls are more appropriate than food frequency or usual intake questionnaires [27] for young children. A similar principal applied with respect to activity recalls. In addition our study is limited by the fact that it is a pilot study and as such it includes a small number of participants. The sex and age adjusted energy intake and activity explained only 18% of the variance in BMI. This is likely due to the fact that energy was assessed as frequencies rather than kilo joules. Factors such as pubertal stage that have major impact on BMI were not assessed although this may be less important for blacks than other races [28]. Nevertheless the fact that the macronutrients were within AMDR was similar to the adult diet and that the relationship of BMI to intake and activity was in the expected directions lends credibility to our findings. 

Our findings clearly underline the important role the family plays in shaping the lifestyle factors which affect weight gain and by extension development of the host of chronic diseases that currently plague Barbados and similar developing countries. The challenge is that much of what constitutes negative influences is integral to the charm and culture of these states. Changes that affect one negative component of the obesogenic environment are unlikely to combat cultural influences [29]. The mounting cost of health care cannot however be ignored and so targeting specific high impact areas may best serve Barbados and the wider Caribbean with respect to stemming childhood obesity. Adults tended to promote sedentary rather than active behaviours at home. This is an area that is opportune for research as little is known about parent-child interactions and activity. In addition, our finding that child influenced by school-based activity was a strong predictor of body size suggests that internal motivation is likely to be an important factor in promoting activity and this is likely highly influenced by adults at home. Importantly activities selected at home did not correlate to body size suggesting perhaps that the home may not provide either physical facilities or a nurturing environment. Clearly both home and school are important areas for preventive intervention research. 

## 5. Conclusions

Our data show that childhood obesity prevention recommendations and research should focus on local practices that promote acquired tastes for excess sugar and culture-specific parent-child interaction regarding physical activity. Child influenced by school-based activity interventions is an important area for preventive intervention research.

## Figures and Tables

**Table 1:** Mean daily macronutrient intake among participants of the pilot of the Barbados Children's Health and Nutrition Study (BCHNS) compared to AMDRs.

	Males (*n* = 29)		Females (*n* = 33)		Total (*n* = 62)	
	Mean	±Standard error	Mean	±Standard error	Mean	±Standard error
Energy (kcal)	2017	66	1732	81.1	1865	54.4
Carbohydrate (g)	282	11.4	259	11.9	270	8.3
% AMDR*	56		59.9		57.9	
Protein (g)	74	2.3	59	3.7	66	2.3
% AMDR*	14.7		13.7		1402	
Fat (g)	67	2.3	53	3.5	60	2.3
% AMDR*	**30**		**27.7**		**28.9**	

^∗^AMDR: acceptable macronutrient distribution ranges [1].

**Table 2:** Comparison of the mean and median daily energy and nutrient intake of children aged 9–11 years: The Barbados Children's Health and Nutrition Study Pilot (BCHNS)^a^.

	Boys				Girls			
	RDA	Mean	SE	Median	RDA	Mean	SE	Median
Energy (kj)	8660–10250	8437	339	8453	7636–8640	7245	276	7126
Energy (kcal)	2070–2450	2016	81	2020	1825–2065	1731	66	1703
Proteins (g)	27–45	74.3	3.7	68.2	27–45	59.2	2.3	57.9
Carbohydrates (g)	—	282.3	11.9	279.8	—	259.4	11.4	244.7
Total fat (g)	—	67.3	3.5	64.4	—	53.4	2.4	51.1
Saturated fat	—	23.5	1.3	22.3	—	19.1	1.0	19.3
Sugar (g)		130.4	7.8	121.9		133.4	7.8	127.5
Fibre (mg)	27–40	12.7	.8	11.5	27–40	11.3	.6	11.0
Vitamin A (*μ*g)	400–500	5798.3	846.1	4682.5	400–600	3837.9	595.8	2142.2
Vitamin D (*μ*g)	5	1.2	.3	.8	5	1.2	.2	.7
Calcium (mg)	600–700	659	39	670	600–700	552	28	551
Sodium (mg)	400–500	3243	158	3105	400–500	2611	116	2582
Iron (mg)	10–12	12.2	.5	12.8	10–15	10.1	.4	9.5
% Energy from total fat	10–15	29.9	.8	30.9	10–15	27.7	.7	27.8
% Energy from proteins	10–15	14.8	.4	14.7	10–15	14.0	.5	13.2
% Energy from saturated fat	<10	10.5	.4	10.6	<10	10.0	.4	9.4
% Energy from sugar	<10	25.8	1.1	25.9	<10	30.4	1.1	31.1
% Energy from carbohydrates	55–60	56.1	1.1	56.0	55–60	59.7	.8	60.4

^a^Intakes were calculated from three 24-hour dietary recalls two weekdays and one weekend day.

RDA: Recommended dietary allowance.

**Table 3:** Nutrient composition of typical lunch provided by the Barbados School Meals Service.

Source	Energy (kcal)	Protein (g)	Carbohydrate (g)	Fat (g)	Sodium (mg)	Calcium (mg)	Sugar (g)	Saturated fat (g)	Iron (mg)
Sample school lunch^a^	519	16	73	20	1323	95	42	6	2.2
Sample meal from Barbados recommendations^b^	601	41	78	15	579	423	31	5.8	5.7

^a^A sample lunch taken from The Barbados School Meals Service Menu.

^b^A sample lunch presented for children over 14 years in the food-based dietary guidelines for Barbados [14].

**Table 4:** The most commonly consumed beverages and foods reported in the 24-hour dietary recalls among 62, 9–11 years old Barbadian children participants of the pilot of the Barbados Children's Health and Nutrition Study (BCHNS).

Food item	Number of times reported
Sugar-sweetened aerated beverages	163
Fruit drinks	148
Water	143
Salty snacks (chips, cheese sticks, etc.)	101
Rice	88
Chocolate tea	84
Sliced breads	69
Chicken, roasted or fried	61
Macaroni pie	61
Colloquial drinks	52
Salt bread	51
Cheese (natural and processed)	46
Peas and rice	43
Breakfast cereals, cold	39
Corned beef	38
Salt fish	38
Milk, plain	37
Milk, flavored	35
Herbal tea	33
Cake	33

**Table 5:** Reported activities by type, location, and person who initiated activity, among participants of the pilot of the Barbados Children's Health and Nutrition Study (BCHNS).

Type of activity	Location	Child selected		Adult selected		Total	
		*n*	Percent of total activity	*n*	Percent of total activity	*n*	Percent of total activity
Active	School	30	4.8%	21	3.4%	51	8.1%
	Home	155	24.9%	109	17.5%	264	42.4%
Subtotal active		185*	29.7%	130*	20.9%	315	50.5%
Sedentary	School	2	0.3%	1	0.2%	3	0.5%
	Home	165	26.5%	140	22.5%	305	49.0%
Subtotal sedentary		167**	26.8%	141**	22.7%	308	49.5%

Total		325**	56.5%	271**	43.5%	623	100%

^∗^ *P* = 0.002; ^∗∗^ *P* < .001.

**Table 6:** The age- and sex-adjusted relationship of energy intake and school-based self-selected activities to BMI among 9–11 year old Barbadian children, participants of the pilot of the Barbados Children's Health and Nutrition Study (BCHNS).

	*B*	S.E	*P*	Adj. *R*2	Δ*R*2
(Constant)	8.077	11.276	0.477		
Sex	−0.962	1.347	0.478		
Age	0.862	1.051	0.420	0.022	0.055
Energy	0.003	0.002	0.066	0.046	0.039
School-based self-selected active activities	−2.966	1.206	0.017	0.125	0.090

Adjusted for current age and sex (males = 0 female = 1).

## References

[1] Foster C, Rotimi C, Fraser H (1993). Hypertension, diabetes, and obesity in Barbados: findings from a recent population-based survey.. *Ethnicity & Disease*.

[2] Cooper R, Rotimi C, Ataman S (1997). The prevalence of hypertension in seven populations of West African origin. *American Journal of Public Health*.

[3] Gaskin PS, Broome H, Alert C, Fraser H (2008). Misperceptions, inactivity and maternal factors may drive obesity among Barbadian adolescents. *Public Health Nutrition*.

[4] Ramsey F, Demas N, Trotter P (1986). *The National Health and Nutrition Survey of Barbados, 1981*.

[5] Fernandez M A Snapshot of childhood overweight and obesity in Barbados.

[6] Sharma S, Cao X, Harris R, Hennis AJM, Wu SY, Leske MC (2008). Assessing dietary patterns in Barbados highlights the need for nutritional intervention to reduce risk of chronic disease. *Journal of Human Nutrition and Dietetics*.

[7] Prochaska JJ, Sallis JF, Griffith B, Douglas J (2002). Physical activity levels of barbadian youth and comparison to a U.S. sample. *International Journal of Behavioral Medicine*.

[8] ASPE (2011). Childhood obesity. *Report of the Assistant Secretary for Planning and Evaluation*.

[9] Beckles HM (1989). *Corporate Power in Barbados The Mutual Affair Economic Injustice in a Political Democracy*.

[10] Mokdad AH, Serdula MK, Dietz WH, Bowman BA, Marks JS, Koplan JP (1999). The spread of the obesity epidemic in the United States, 1991–1998. *Journal of the American Medical Association*.

[11] Lohman TG, Roche AF, Martorell R (1988). *Anthropometric Standardization Reference Manual*.

[12] (2005). *Dietary Reference Intakes for Energy, Carbohydrate. Fiber, Fat, Fatty Acids, Cholesterol, Protein, and Amino Acids*.

[13] CFNI (1994). *Recommended Dietary Allowances For the Caribbean*.

[14] The National Nutrition Centre (2010). *Food Based Dietary Guidelines for Barbados National Nutrition Centre*.

[15] Ainsworth BE, Haskell WL, Whitt MC (2000). Compendium of physical activities: an update of activity codes and MET intensities. *Medicine and Science in Sports and Exercise*.

[16] Cole TJ, Bellizzi MC, Flegal KM, Dietz WH (2000). Establishing a standard definition for child overweight and obesity worldwide: international survey. *British Medical Journal*.

[17] The National Nutrition Centre (2010). *Nutritious and Healty Foods in Schools; Nutritional and Practical Guidelines for Barbados*.

[18] https://www.cia.gov/library/publications/the-world-factbook/.

[19] Thorpe LE, List DG, Marx T, May L, Helgerson SD, Frieden TR (2004). Childhood obesity in New York City elementary school students. *American Journal of Public Health*.

[20] Freedman DS, Khan LK, Serdula MK, Ogden CL, Dietz WH (2006). Racial and ethnic differences in secular trends for childhood BMI, weight, and height. *Obesity*.

[21] Räsänen M, Niinikoski H, Keskinen S (2001). Nutrition knowledge and food intake of seven-year-old children in an atherosclerosis prevention project with onset in infancy: the impact of child-targeted nutrition counselling given to the parents. *European Journal of Clinical Nutrition*.

[22] Papalia D, Olds S, Feldman R (2005). *Human Development*.

[23] (2002). *Dietary Reference Intakes for Energy, Carbohydrate, Fiber, Fat, Fatty Acids, Cholesterol, Protein and amino Acids*.

[24] Strasburger VC, Mulligan DA, Altmann TR (2011). Children, adolescents, obesity, and the media. *Pediatrics*.

[25] Must A, Tybor DJ (2005). Physical activity and sedentary behavior: a review of longitudinal studies of weight and adiposity in youth. *International Journal of Obesity*.

[26] Ma Y, Olendzki BC, Pagoto SL (2009). Number of 24-hour diet recalls needed to estimate energy intake. *Annals of Epidemiology*.

[27] Baxter SD, Smith AF, Litaker MS (2004). Recency affects reporting accuracy of children’s dietary recalls. *Annals of Epidemiology*.

[28] Kaplowitz PB, Slora EJ, Wasserman RC, Pedlow SE, Herman-Giddens ME (2001). Earlier onset of puberty in girls: relation to increased body mass index and race. *Pediatrics*.

[29] Gordon-Larsen P, Adair LS, Popkin BM (2003). The relationship of ethnicity, socioeconomic factors, and overweight in U.S. adolescents. *Obesity Research*.

